# Cnidarian Immunity and the Repertoire of Defense Mechanisms in Anthozoans

**DOI:** 10.3390/biology9090283

**Published:** 2020-09-11

**Authors:** Maria Giovanna Parisi, Daniela Parrinello, Loredana Stabili, Matteo Cammarata

**Affiliations:** 1 Department of Earth and Marine Sciences, University of Palermo, 90128 Palermo, Italy; daniela.parrinello@unipa.it; 2 Department of Biological and Environmental Sciences and Technologies, University of Salento, 73100 Lecce, Italy; loredana.stabili@irsa.cnr.it

**Keywords:** cnidarians, innate immunity, Anthozoan, bioactive molecules, inflammatory response

## Abstract

Anthozoa is the most specious class of the phylum Cnidaria that is phylogenetically basal within the Metazoa. It is an interesting group for studying the evolution of mutualisms and immunity, for despite their morphological simplicity, Anthozoans are unexpectedly immunologically complex, with large genomes and gene families similar to those of the Bilateria. Evidence indicates that the Anthozoan innate immune system is not only involved in the disruption of harmful microorganisms, but is also crucial in structuring tissue-associated microbial communities that are essential components of the cnidarian holobiont and useful to the animal’s health for several functions including metabolism, immune defense, development, and behavior. Here, we report on the current state of the art of Anthozoan immunity. Like other invertebrates, Anthozoans possess immune mechanisms based on self/non-self-recognition. Although lacking adaptive immunity, they use a diverse repertoire of immune receptor signaling pathways (PRRs) to recognize a broad array of conserved microorganism-associated molecular patterns (MAMP). The intracellular signaling cascades lead to gene transcription up to endpoints of release of molecules that kill the pathogens, defend the self by maintaining homeostasis, and modulate the wound repair process. The cells play a fundamental role in immunity, as they display phagocytic activities and secrete mucus, which acts as a physicochemical barrier preventing or slowing down the proliferation of potential invaders. Finally, we describe the current state of knowledge of some immune effectors in Anthozoan species, including the potential role of toxins and the inflammatory response in the Mediterranean Anthozoan *Anemonia viridis* following injection of various foreign particles differing in type and dimensions, including pathogenetic bacteria.

## 1. Introduction

Cnidarians are phylogenetically basal aquatic animals within the Metazoa, with radial symmetry and the first level of tissue organization. They are evolutionarily early-diverged Metazoa, some of whom can live for hundreds of years, which suggests that they are potentially exposed to some pathogens on many occasions during their lifespans [1]. It is difficult to explain how these long-lived organisms have done so well with only an innate immune system as the protective mechanism against infectious agents.

Cnidarians are of great interest since they apply many of the same cellular pathways involved in innate immunity in mammals. They have a surprising amount of immune complexity, as they contain innate immune components that are lacking in other basal invertebrate groups [2].

In addition to pathogen recognition, the cnidarian innate immune system has a role in managing beneficial microbes and supporting mutualistic microbial symbioses. It regulates the maintenance of symbiosis and takes on the task of discerning between pathogens that need to be cleared versus beneficial symbiotic microbes [3].

**Figure 1:**
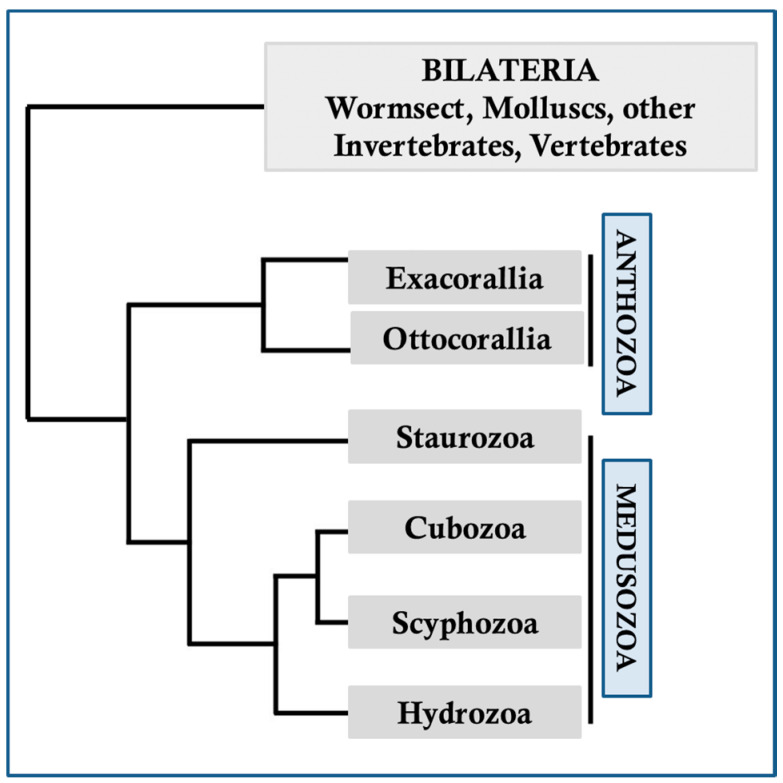
Cnidarian evolutionary history based on rRNA phylogenies.

Anthozoa is the most speciose class of Cnidaria, including the subclasses Hexacorallia and Octocorallia, which comprise hard corals or anemones and soft corals and gorgonians (Figure 1).

It is well known that Anthozoan epithelia are colonized by a community of associated microorganisms that influence animal development and host fitness. Epithelium colonization is also determined by the availability of nutrients, and competitive interactions for the colonization of the animal substrate by different symbiotic bacterial strains.

Several immune response genes have been conserved from cnidarians to vertebrates [4,5,6,7,8].

The immune system of cnidarians is based on self/non-self-recognition and it is comprised of four major functions: the immune recognition regulated by membrane pattern recognition receptors (PRRs) that bind to molecular patterns in pathogens (MAMPs); activation of a range of transcription factor; intracellular signaling cascades leading to gene transcription and ultimately protein translation; endpoints of release of the proteins and molecules that eliminate the threat and mitigate self-harm, kill the pathogen (antibacterial molecules, reactive oxygen molecules, antioxidant, phagocytosis, and other cellular activities), and defend self by maintaining homeostasis.

These effectors are antibacterial molecules, reactive oxygen molecules, antioxidant, and cellular activities. Finally, the specimens must also close any wounds and regenerate the destroyed tissues.

The epithelial cells play a fundamental role in immunity as they display phagocytic activities and secrete mucus, which acts as a physicochemical barrier preventing or slowing down the proliferation of potential pathogens [9,10].

The mucus contains several protector factors, including serine protease inhibitors with bactericidal activity and antimicrobial peptides (AMPs) [11,12].

The Anthozoans can distinguish self from non-self like tissues contiguous conspecifics [13]. This allorecognition and xenorecognition capability with related killing mechanisms has been established in Hydrozoans and Anthozoans [14,15,16]. In the colonial cnidarians, the allorecognition process limits the fusion with genetically different individuals as well as the parasitic germ line, starting with a contact avoidance response and culminating in the usage of nematocysts.

As invertebrates who rely on the innate component of immunity, they should not be able to develop resistance toward a particular pathogen [17]. However, a form of immunological memory has long been suggested in cnidarians, based on tissue allo- and xeno- transplants [18]. It is true that the mechanisms underlying recognition may differ from those involved in the functioning of the defense system.

Further evidence of immunological memory derives from classes of receptors that recognize molecular models of different pathogens [19]. This means that Anthozoans can recognize the difference between pathogenic types, responding with specific binding receptors to acquire memory for a specific pathogen.

Here, we reported the current state of knowledge about cellular and molecular immune repertoire in Anthozoan species and the inflammatory responses in *Anemonia viridis* (Cnidaria: Anthozoa) following bacterial injection and pathogenetic invasions with bacteria such as *Escherichia coli* and *Vibrio alginolyticus.* These belong to different genera and are of diverse shape and dimension. 

The currently accepted taxonomic scheme subdivides Cnidaria into two main assemblages: Anthozoa (Hexacorallia and Octocorallia) with a reproductive polyp and the absence of a medusa stage and Medusozoa (Cubozoa, Hydrozoa, Scyphozoa, and Staurozoa) that usually possess a reproductive medusa stage.

## 2. Allorecognition in Anthozoans

In marine colonial invertebrates (including members of the phylum Chordata), allorecognition may be essential for survival as a response to selection pressures [18]. In vertebrates, allorecognition and self-tolerance are the result of a major histocompatibility complex (MHC)-based recognition event, critical to adaptive immunity.

In many invertebrate colonies that share alleles of specific histocompatibility, loci (Alr 1, Alr 2) generally merge to create a chimeric colony, while individuals who do not participate in this sharing are aggressively rejected. In cnidarians, the Alr1 and Alr2 loci encode membrane-bound molecules with immunoglobulin-like domains not homologous to those found in basal chordate [20].

In Anthozoans, such as *Montipora verrucosa*, branches within a colony can easily join, while branches of genetically diverse individuals do not fuse [21]. *Hydractinia symbiolongicarpus* is a cnidarian model studied in the context of allorecognition—a hydroid feeding on gastropods inhabited by hermit crabs along the intertidal areas of the east coast of North America. In this species, fusion occurs when the colonies share one of two, or both, alleles of two loci. By contrast, the rejection reaction, characterized by the non-adhesion and the discharge of the nematocysts, occurs when the matched partners do not share any of these alleles. The *Hydractinia* colonies are composed of polyps, the bowels of which are connected to each other by a network of gastrovascular channels. These channels then extend out of the carpet to grow along the surface of the shell, forming stolons. When the stolons encounter an incompatible colony, they differentiate into “hyperplastics”, loaded with cnidocytes, growing on the opposite colony and releasing cnidocytes until the death of the incompatible colony. In the case of compatibility, a chimeric colony is formed [22]. The Alr1–Alr1 and Alr2–Alr2 interactions modulate self/non-self-discrimination, and their long cytoplasmic tails (200–250 amino acids) contain motifs reminiscent of sites for kinase-mediated phosphorylation.

In *Hydractinia,* Mokady & Buss (1996) showed that these two closely related loci for recognition are contained in a single chromosomal region [23,24]. Genomic analysis of the region corresponding to the Alr2 locus led to the identification of a transmembrane protein, CDS7, which has three extracellular polymorphic domains [25]. The CDS7 domain polymorphism represents an element of histocompatibility as a probable candidate responsible for the recognition in basal metazoans. Research efforts on the identification of molecules involved in recognition in solitary cnidarians, such as *Hydra*, were not successful thus far [26].

## 3. Symbiosis and Immunity

The association of sea anemones with photosynthetic algae of the genus *Symbiodinium*, collectively called zooxanthellae, provides mutualistic relationships [27]. The cnidarians supply nutrients to the zooxanthellae, as well as carbon dioxide derived from the digestion of the preys; in turn, the symbiont photosynthetic zooxanthellae produce sugars, lipids, and oxygen that support energy production for the metabolic activity of the Anthozoan, associated with microbial communities [28]. The unicellular algae are in gastrodermal cells within host-derived membrane vesicles called symbiosomes that arise during the symbiotic process of the cnidarian host [29]. The symbiotic relationship is useful for studying the evolution of the various groups. Some symbionts’ taxa have a notably wide geographical distribution and are associated with multiple species of hosts. Others, however, show a restricted geographical distribution [30,31].

Transcriptomic studies have shown that homologs of genes involved in the defense system and inflammation as mediators of oxidative stress, serine protease, or immunity transcription factor NF-κB are downregulated by the establishment of symbiosis and upregulated when lacking in symbiotic cnidarians [32].

Symbiosis is recognized as a non-harmful infection, and host defense responses are controlled until environmental conditions are optimal for the survival of autotrophic and heterotrophic organisms; the role of bacterial communities has yet to be explained [33].

Bleaching is a host-innate immune response to a compromised symbiont, much like innate immune responses in other host–microbe interactions [34].

Although the downregulation of immunity appears to be a necessary condition to symbioses, evidence from certain bleached corals suggests that immunity genes are less expressed [35]. In this respect, some Anthozoan specimens losing symbionts resulted in increased susceptibility to pathogen-induced diseases greater than that of symbiotic specimens. Pollution induces the symbiosis breakdown and the zooxanthellae can be expelled, exposing the specimens to the greatest risk of mortality.

For some time, people have been wondering if autophagy and apoptosis have been working together to induce loss of symbiosis during heat stress-induced bleaching [36]. It has been proposed that the Rab GTPase, a regulating lysosome–phagosome fusion gene family, plays key roles in maintaining symbiosis with host gastrodermal cells [37].

The gene encoding Rab32, a regulator of the lysosomal enzyme recruitment to phagosome, is involved in the exclusion and maintenance of symbionts [38]. In *Aiptasia pulchella*, the upregulation of Ap-Rab7 during heat stress induces the phagosomal–lysosomal fusion of the Symbiodiniaceae-containing vacuoles, leading to the loss of symbiosis.

The symbiotic relationship is useful for studying the evolution and biogeography of the various zoological groups. Some symbionts taxa in fact have a wide geographical distribution and are associated with multiple species of hosts [39].

## 4. Interaction with Microbial Communities 

Cnidarians host a variety of microbes including many bacterial species and viruses [40,41]. Identification of the unique role of the microbiota in eukaryotic host development, and their response to environmental perturbations, has led to the definition of a “holobiont”. For Anthozoans, too, it describes a complex organism in which multiple components can evolve together as symbiotic entity [42]. These multi-partite symbiotic organisms are formed by polyp animals, zooxantellae, and microbial assemblages associated with polyps and photosynthetic symbionts. 

There is increasing evidence to indicate that the cnidarian innate immune system is not only involved in the disruption of harmful microorganisms, but is also crucial in structuring tissue-associated microbial communities that are essential components of the holobiont, and useful to the animal’s health [43].

Cnidarians have many microorganisms associated (epibiotic or symbiotic) with their tissues. As reported by Tinta et al. [44], early reports on microbes associated with cnidarians resulted as corollary observations, whereas primary targets of research were cnidarians. Later studies, focusing on the relationships between microbes and their host organisms, addressed more specific issues on the composition and ecological role of cnidarian-associated microbial communities. These studies dealt with issues on the role, ecology, and composition of microbial communities associated with cnidarians, the mechanisms at the base of these interactions, and the nature of the relationships established between cnidarians and their associated microbiome. On account of these considerations, researchers were focused on the microbial counterpart of the jellyfish–microbe associations, in order to assess the diversity of microbial communities associated with different jellyfish species from various ecosystems and with their different life stages and body compartments. Further studies concerned bacteria associated to outer surfaces of cnidarian epithelia in different taxa and life stages showing their involvement in a number of important potential roles including antibiotics synthesis, nitrogen fixation, organic compounds decomposition, primary defense against pathogens, or modulation of contractile activities.

The microbial communities associated with the semeostome jellyfish *Aurelia aurita*, the rhizostome jellyfish species *Mastigias papua*, *Cotylorhiza tuberculata*, and *Rhizostoma pulmo* and the box jellyfish *Tripedalia* cf. *cystophora* were analyzed in different studies, identifying jellyfish as a host of bacterial associates. The microbiome associated with different life stages of *A. aurita* (polyp, strobila, ephyrae, juvenile, and adult medusae) was examined by Weiland-Bräuer et al. [45]. The authors also considered different compartments of the adult medusae (exumbrella, mucus, and gastric cavity) and compared the microbiome associated with specimens (polyp stage) collected in different geographic sites. In particular, the microbial community of jellyfish *A. aurita* seems to be strictly host-specific and different from the bacterioplankton suspended in the surrounding water column. In *A. aurita* adult medusa, the microbiota of different compartments exhibit significant differences, showing body part-specific bacterial colonization with a mucus-associated bacterial composition that is more variable compared to bacteria living in the gastric cavity, likely thanks to trapping properties of mannose and mucine glycan components of mucus. Moreover, Kos Kramar et al. [46] checked the bacterial community associated with the moon jellyfish *Aurelia solida*. In particular, different body parts (exumbrella surface, oral arms, and gastric cavity) were analyzed for the bacterial community diversity. Furthermore probable differences in medusa-associated bacterial community structure during the jellyfish population peak and the senescent stage when the bloom ended were evaluated. The authors concluded that microbiota associated with moon jellyfish were different from microbial assemblages in the surrounding seawater and differed between different body compartments. The microbiota in the gastral cavity of medusa Betaproteobacteria (*Burkholderia*, *Cupriavidus*, and *Achromobacter*) prevailed, by contrast, over those on the ‘outer’ body parts of Alphaproteobacteria (*Phaeobacter*, *Ruegeria*) and Gammaproteobacteria (*Stenotrophomonas*, *Alteromonas*, *Pseudoalteromonas* and *Vibrio*). During the senescent phase, at the end of the jellyfish bloom, the bacterial community resulted changed in the structure with an increase of Gammaproteobacteria, wholly *Vibrio*. On the basis of these results, it was hypothesized that the jellyfish-associated bacterial community might play an important role for the host. Analyzing the composition of the microbiome associated with jellyfish mucus, Tinta et al. [44] concluded that Gammaproteobacteria (mainly *Pseudoalteromonas* and *Vibrio*) are abundant, but to some extent also Alphaproteobacteria (*Phaeobacter*, *Rugeria*, and *Roseovarius*). These bacteria, due to their capability to synthesize antimicrobial compounds when attached to live or inert surfaces, were previously recognized as significant players in the host defense towards pathogens and fouling organisms from the surrounding environment. *Rhizostoma pulmo*-associated microbiota were investigated in three distinct compartments, namely umbrella, oral arms, and the mucus secretion [47,48]. Actinobacteria, Bacteroidetes, Chlamydiae, Cyanobacteria, Deinococcus-Thermus, Firmicutes, Fusobacteria, Planctomycetes, Proteobacteria, Rhodothermaeota, Spirochaetes, Tenericutes, and Thaumarchaeota were the phyla isolated from all the three *R. pulmo* compartments in the sampling times. In particular, the main genera Mycoplasma and Spiroplasma, belonging to the class Mollicutes (phylum Tenericutes), have been identified in all three jellyfish compartments. Microorganisms associated with mucus are characterized by great diversity and the mucus appears to be the compartment mainly rich in bacteria compared to the oral arms and the umbrella.

In Anthozoans microbial agents fulfill several functions, including regulation of metabolism, immune defense, development, and behavior. Bacteria associated with Anthozoan tissues can indeed fix nitrogen, digest complex polysaccharides, and produce antibiotics to prevent infection with pathogens. In turn, *Symbiodinium* produces dimethylsulfoniopropionate (DMSP) as osmolite, antioxidant agents [49], and a nutrient source for associated bacteria [50].

A certain specificity was also found in the Anthozoan-bacteria association; Porporato et al. [51], for example, described the bacterial communities associated with *Pennatula phosphorea* and *Pteroeides spinosum*. Results from this study showed the occurrence of species-specific coral-associated bacteria since *P. phosphorea* and *P. spinosum* host distinct bacterial communities. Moreover, since in the same pennatulid species, only a few phylotypes were shared between mucus and tissues, it was hypothesized that there might exist a microhabitat partitioning between the associated microbial communities as already described for jellyfish. In the case of *P. phosphorea*, the communities associated with both tissues and mucus were characterized by the predominance of Alphaproteobacteria. Conversely, the Alphaproteobacteria prevailed in the mucus layer of *P. spinosum* and the tissue library was dominated by the Gammaproteobacteria and Mollicutes. Bacterial isolates belonging to *Vibrio* spp., mainly obtained from coral mucus, showed an antibacterial activity against indicator organisms, indicating a protective function of the coral-associated bacterial communities as in the case of jellyfish.

On account of these evidences the ability of cnidarians to control production and composition of a mucosal matrix and its associated bacteria can represent an important part of immunity [52]. Mucus indeed contains many microorganisms and bacteria, and the *Vibrio* genus particularly prevails in the cultivable bacterial isolates from the mucus of several other Anthozoans [53].

Some mucus bacteria producing antimicrobial compounds contribute to competition in space and nutrition with the host potential pathogenic bacteria. Due to these features, mucus and its components have interesting biotechnological implications.

The specific interactions of the microbial colonization of mucosal surfaces are still unknown. The “Coral Probiotic Hypothesis” [54] affirms that *Pseudoalteromonas* sp. can be considered as “probiotic” to corals, taking part in the coral holobiont defense against bacteria, placing a different critical perspective on the host–microbe interactions.

Calow [55] indicated that the differences in mucus biochemical composition influence the attack of several microbial agents that use exoenzymes to degrade mucoid polymers. Microbes themselves can transform dissolved and particulate matter into living matter, attracting other predatory organisms.

It also appears that change in the density of bacterial communities may influence animal health and, consequently, their sensitivity to disease [54].

Recent reports of the succession of the microbial communities associated with the developmental stages of *Porites astreoides* [56] and the discovery of the potentially beneficial functions in α-Proteobacteria and strains of *Marinobacter* [16] lend further support to the hologenome evolution hypothesis indicating that in colonial symbiotic organisms, the ‘hologenome’, deriving from all the members of the holobiont, acts as a single unit of evolution, with faster evolving micro-organisms providing the plasticity to adapt to the changing environment [57]. 

Controversies still surround this aspect of the Anthozoans, as it is not yet clear how some corals are not susceptible to the pathogens that have caused diseases in the past [58]. Furthermore, the natural selection for disease-resistant coral genotypes seems to have acted, which could explain the loss of virulence by the bacterial specimens. On the other hand, disease has been increasing over the last few decades in response to elevated sea surface temperatures and anthropogenic stressors causing bleaching and eventual death of the tissue [9,59]. Coral bleaching, for example, occurs when the balance between the different components of the holobiont is destroyed and immune cascades are activated, which lead to Nuclear factor-κB (NF-κB) protein levels up-production, involved in an elaborate system that enables a response to environmental changes. The elimination of the symbiont from Anthozoans tissues is regulated by a variety of mechanisms such as exocytosis, host cell detachment, and apoptosis [60].

## 5. Repertoire of Immune Receptors Signaling Pathways (PRRs)

The sessile Cnidaria lack a protective barrier and are thus exposed to external abiotic and biotic factors. They have consequently developed defense mechanisms based on a wide range of pattern recognition receptors (PRRs) that act as a physical-chemical barrier [61]. Pathogen recognition occurs through the detection of molecular models associated with microbes (MAMP) as lipopolysaccharides or lipoteichoic acid [62] and host-derived damage-associated molecular patterns [17,63] using a wide range of PRRs. These are also involved in maintaining homeostasis between the host and the epithelium microbiota.

These bindings of germ line-encoded receptors to MAMPs activate complex downstream signaling pathways that result in the expression, via transcription factors or serine protease cascades, of immune genes that initiate the proper effector responses, such as opsonization, phagocytosis, lysis, or the production of antimicrobial peptides [64]. The gene coding for the nuclear transcription factors NF-kB, that activates the expression of genes involved in a broad range of immune processes has been characterized in *N. vectensis* and *A. millepora*, while in *Hydra*, the activation of NF-kB leads to the expression of AMPs [65]. Genes encoding the adaptor protein MyD88 and kinases that participate in the signal delivery, such as IRAK, TRAF and TAK, have also been identified [66]. Components of other signaling transduction pathways triggered by PRRs seems to be conserved in cnidarians such as those involved in the Interferon and ECSIT signaling pathways [2]. Soluble (sPRRs), membrane (mPRRs), and cytoplasmatic (cPRRs) have been characterized in cnidarians. The first two recognize destroyed-self molecular patterns on cell surface and non-self microrganisms. 

Principal cnidarian PRR and MAMP are indicate in Table 1.

### 5.1. Toll-Like Receptors (TLRs) 

Among the PRR family, the Toll-like receptors (TLRs) are glycoproteins embedded within the host membrane which possess domains specific for binding MAMPs. They are proteins composed by an extracellular N-terminal domain having leucine rich repeats (LRRs) responsible for the recognition process, a cysteine-rich domain, a transmembrane domain, and an intracellular Toll/Interleukine-1 receptor (TIR) [62].

Several signaling pathways associated with TLRs have been identified, such as the homologues of the transcription factor NF-κB, which lead to cell death and the homologues of the myeloid differentiation primary response adaptor protein (MyD88), which activates downstream signaling pathways. 

TLRs have ancient roles in NF-κB signal transduction, pathogen detection, and development, thus providing molecular insights into how invertebrates basal phyla may respond to pathogens.

In addition to NF-κB signaling, TLR receptors can activate mitogen-activated protein kinase (MAPK) resulting in activation of the immune signaling pathways and appropriate effector responses [63]. The known pathway homology indicates that the Toll/TlR receptors are highly conserved.

Since the invertebrate immunity is innate and non-specific, the effector responses can be activated in relation to serine protease cascades and redox signaling [64] without gene transcription.

In *Hydra magnipapillata*, four TLR-domain proteins HmMyD88-1 and HyMyD88-2 are related to the downstream MyD88. The other two Hydra TIR-domain proteins, HyTRR-1 and HyTRR2, are likely to be pathway initiator receptors, and they lack the LRR-domains [2].

Among the Anthozoa, transcriptomic and genomic studies have resulted in the discovery of many more putative TLRs proteins in *Nematostella vectensis* [2] and *Gorgonia ventalina* [78]. One *N. vectensis* TLR (Nv-TLR) is also expressed in some cnidocytes, and it is able to express Nv-NF-κB, another innate immune pathway to engulf the coral pathogen *Vibrio coralliilyticus* [61].

Five TLR-domain proteins have been identified from the predicted structures in *Nematostella vectensis*. The structure of NvMyD88 appears similar to the HmMyD88-1 and 2 and initiates the transmission of intracellular signals leading to the translocation of transcription factors from the NF-kB family [79].

Other identified predicted TLR-domain protein structures include immunoglobulin (Ig) domains which act in cell–cell recognition, resulting in similar architecture to mammalian interleukin receptors (ILR) but form a distinct clade away from the higher vertebrate structures.

In *Acropora digitifera*, several TLRs and ILRs have been identified, suggesting the immune recognition repertoire in this coral species is different than that of *Nematostella*. Downstream components of the Toll/TLR pathways were also described from the EST/genome analysis, such as those associated with the c-Jun N-terminal kinases (JNK)/Mitogenactivated protein kinase (MAPK) pathway and NFkB transcription that can lead to cell death [80].

### 5.2. NOD-Like Receptors (NLRs)

The structure of NLRs consists of a C terminal LRR domain for microbe pattern recognition, an intermediary NOD domain for nucleotide binding and modulation of NLR activity, and an N-terminal effector domain. The family of NOD-like receptors (NLRs) that function as cytosolic receptors form a signaling scaffold to activate an inflammatory cascade. In the corals *Acropora digitifera* and *Pseudodiploria strigos*, an array of NOD-like receptor proteins has been detected. Like TRLs, the NLR activation triggers multiple proinflammatory signaling pathways, which result in the inhibition of the pathogens [81,82].

PRRs can also recognize damage-associated molecular patterns (DAMPs), which are self-molecules or debris from altered cells, and they can trigger the response mediated by the immunological effectors. These are substances inside the cells which are not easily detected by the surrounding cells, but are released in the case of danger or injury to signal the presence of a threat to the healthy cells forming the surrounding tissue.

### 5.3. Lectins

Lectins act as PRRs. A variety of lectins have been described in cnidarians [81,83,84] involved in opsonization activity and activation of the complement cascade. The complement lectin pathway, in fact, has been detected among the cnidarians, in which lectin binds to the sugar present on the pathogen surface, activating mannose-binding lectin associated serine proteases (MASPs), C2 and C4-like proteins up to formation of the C3 complex. After the complex formation, membrane attack complex perforin (MACPF) effector proteins are secreted to realize a hole in the microbial membrane. The resulting effector is the lysis of the pathogen. One lectin common to several groups of cnidarians is the tachylectin [85], initially isolated from *Tachypleus tridentatus*, which has experimentally shown antimicrobial activity and recognition of MAMP as LPS and peptidoglycans.

MAMP-PRR interactions occur during the establishing or in the maintenance of symbiosis.

Cnidarian lectins that could participate in MAMP-PRR interactions in symbiosis establishment have been identified by a genomic approach. A d-Galactose-binding lectin, SLL-2, was purified from the octocoral *Sinularia lochomodes*, sequenced, and found by immunolocalization to occur surrounding symbiotic dinoflagellates in the gastrodermis. Lectin-binding patterns varied between different *Symbiodinium* types, suggesting a complex surface glycome that varies between types. N-Acetyl and mannose residues are well-characterized MAMPs that bind to mannose-binding lectins and ficolins, respectively [86].

Millectin, involved in both symbiont recognition and an innate immune response to bacteria, has been described as primary PRR in the coral *Acropora millepora* and it is expressed in both nematocysts and the gastroderm. This lectin is expressed around intracellular symbiont cells in vivo and some domains are similar to the vertebrate mannose binding lectin (MBL) [75,83]. 

*Hydractinia symbiolongicarpus* and other cnidarians express one rhamnose-binding lectin (RBL) gene, rhamnospondin, which contains multiple PRR-type domains with probable opsonization or agglutination activity [87].

### 5.4. Integrin 

Integrins are trans-membrane alpha beta heterodimers that mediate the interactions between cells and the extracellular matrix ECM [72]. They are involved in multiple immunological cellular processes such as cell migration, differentiation, signal transduction, and wound repair. ECM engagement leads to the activation of focal adhesion kinase (FAK) and to the tyrosine kinases. Three integrin have been identified in *N. vectensis* and two beta subunits have been identified in the hard coral *A. millepora* [2,73].

### 5.5. Other PRRs

A putative immune recognition repertoire of the cnidarians, involved in recognition and response to microbial infection, in addition to the categories discussed, through a variety of molecular methods, has been identified. These are specifically leucine rich repeat (LRR) identified in expressed sequence tags (ESTs) [74], and a transforming growth factor β (TGF-β) receptor [88].

A family of PRRs that recognize LPS from Gram-negative bacteria, leading to the activation of NF-kB pathway, is the lipopolysaccharide (LPS)-binding proteins (LBPs) identified in the genomes of *H. magnipapillata* and *N. vectensis* [2].

The scavenger receptors (SR), characterized by the presence of scavenger receptor cysteine-rich (SRCR) domains, which recognize a wide variety of molecular patterns, have been identified through EST analysis in the corals *M. faveolata* and *A. palmata* [74].

## 6. Molecular Signaling

After the receptors’ activation, the intracellular domains propagation of the signal though the cell is pivotal to the activation of the immune responses. The multitiered kinase pathway have been identified in cnidarians as a component of TLR pathways [2]. They consist of mitogen-activated protein kinases (MAPK) and extracellular signal-regulated kinases (ERK). A derived group of GTPases, a family of hydrolase enzymes, known as GIMAPS, have been identified in the transcriptome of the *Acropora millepora*, where they are upregulated when challenged with bacterial and viral PAMPs [89]. In addition to these signaling pathways, the complement and phenoloxidase-like systems have been documented in Anthozoans.

### 6.1. Melanin Synthesis Pathways Activation 

Melanin synthesis is a critical component of invertebrate immunity during pathogen encapsulation and wound healing process. In arthropods, melanin synthesis is initiated by PRRs that trigger the activation of serine protease cascades, up to the cleavage of the prophenoloxidase (PPO) and the subsequent formation of active phenoloxidase (PO) enzymes [64]. The following proteolytic cascades lead to melanin production. PPOs and POs exist in various isoforms as components of melanin synthesis types including tyrosinase and laccase [12,90], involved in defense from infection and in cuticle formation respectively [91].

The receptors and mechanisms involved in melanin synthesis pathway activation have not been elucidated well for cnidarians [92]. However, genes homologous to those involved in melanin synthesis in other invertebrates have been identified within the genomes or transcriptomes of several Anthozoans [64] and deposits of melanin have been detected biochemically and/or histologically in Anthozoans tissues. 

In *Pocillopora damicornis*, two genes of the prophenoloxidase pathway (prophenoloxidase activating enzyme and laccase) resulted up-regulated after bacteria challenge [93] while in the genomes of *Nematostella vectensis* and *Hydra magnipapillata*, tyrosinase genes have been identified [94].

PO-like activity also seems to be involved in symbiosis; it is upregulated in the coral *Montipora aequituberculata* when subjected to heat stress [95]. Similarly, naturally bleached species had higher ProPO-like activity compared to both healthy and diseased symbiotic corals. Anthozoans also use cellular defense activities to respond to fungal infection during a stressful temperature event. In the sea fan *Gorgonia ventalina*, the granular acidophilic amoebocytes, normally involved in wound repair and histocompatibility, participate in pathogenic response. In this species, injected with the fungal pathogen *Aspergillus sydowii*, an increase in granular amoebocytes has been shown to be adjacent to infection with respect to other areas of mesoglea [96]. However, in naturally infected sea fans, the melanosomes were found to be adjacent to a protective melanin band in relation to the level of infected tissues with fungal hyphae.

Granular amoebocytes containing melanin have been characterized within hard corals. In recent years, the involvement of cells in a post-injury recovery process and an increase in melanin production in *Porites cylindrica* [97]. This suggests the capacity of this coral to respond to localized stressors [90] and indicates the prominent role of increase in melanin cell density in coral immunity.

### 6.2. Complement System 

The complement system is a basal effector mechanism in animals functioning in cytotoxicity, opsonization, regulation of inflammatory responses, and bacterial lysis [98]. The complement system is activated by three parallel proteolytic cascades, known as the classical pathway, the mannan-binding lectin (MB-lectin) pathway, and the alternative pathway. The MB pathway is triggered by mannan-binding lectin, a normal serum constituent that binds some encapsulated bacteria. The alternative pathway is regulated directly by pathogen surfaces, which converge in the cleavage of the Complement component 3 (C3) to generate inflammatory factors and bacterial lysis.

The central molecule in the pathway C3 serves as an opsonin that is recognized by complement receptors to initiate phagocytosis, and activates an inflammatory response [99]. The result is the formation of the membrane attack complex (MAC) that results in microbe lysis and destruction [98]. Complement C3 plays a pivotal role in the innate immune system of mammals. C3 has a unique intra-chain thioester bond that is shared by some complement and non-complement proteins forming a thioester protein (TEP) family [100]. Phylogenetic analysis of TEP family genes in both vertebrates and invertebrates revealed that the TEP family is divided into two subfamilies, the C3 subfamily and the alpha-2-macroglobulin (A2M) subfamily. The establishment of the TEP genes and differentiation of them into the C3 and A2M subfamilies occurred prior to the divergence of Cnidaria and Bilateria, in a common ancestor of Eumetazoa more than 600 MYA [99].

The sequencing of the *Nematostella vectensis* genome revealed two C3, two alternative pathway serine protease Factor B and one mannan-binding protein-associated serine protease (MASP) genes [101]. The discovery of MASP in a cnidarian genome was a surprise, as this gene had previously only been found in a few deuterostome species [102]. C3 has also been reported in several other coral and anemone species including *Swiftia exserta*, *Acropora millepora*, *Acropora digitifera*, *Haliplanella lineata, Porites lobata*, and *Anemonia viridis* [103,104]. There are complement or precursors of the complement pathways, in the form of C3-like thiolester-containing proteins (TEP) that predate the protostome-deuterostome split identified in cnidarians [2,103]. The first C3-like, TEP cDNA to be identified from the gorgonian coral, *Swiftia exserta*, had high conservation to vertebrate C3. There was an overall similarity with mammalian C3, C4, and C5 sequences. Miller et al. [2] described a suite of predicted proteins with a membrane attack complex (MAC) and perforin domains associated with the final phase of the complement cascade, indicating that multiple components from different stages of the complement cascade pathways exist in the cnidarians. 

## 7. Effector Responses

**Figure 2:**
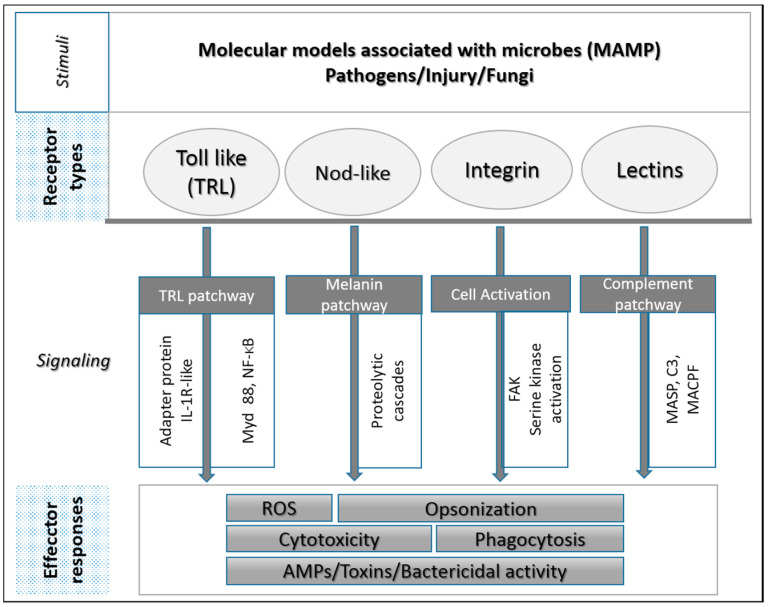
General scheme of the main invertebrate immunity components identified within Anthozoans. TLR, TOLL-like receptor; C3 Complement protein; IL-1Rs, Interleuchin-like; MyD88, myeloid differentiation primary-response protein 88; transcription factors NF-κB; C3, Complement protein; MASPs, mannose binding lectin-associated serine proteases; MACPF, Membrane-attack complex–perforin protein; FAK, focal adhesion kinases; AMPs, antimicrobial peptides; ROS, reactive oxygen species.

After binding of PRRs-MAMPs, the signaling molecules and transcription factors communicate the signal within the cell, activating the intracellular signaling component of immunity. The endpoints of the signaling cascades are the immunological effectors, proteins and molecules that kill the pathogens (Figure 2). A diversity of effector responses has been identified in Anthozoans. These are a surface mucus layer (SML), a polysaccharide protein lipid that limits the pathogens invasion, the antimicrobial peptides, and the reactive oxygen species (ROS) released as byproducts of host and symbiont metabolic processes. The specimens possess also circulating populations of amoebocytes that can migrate to sites of injury or infection and phagocytose foreign cells resulting in release of proteolytic enzymes and free radicals.

### 7.1. The Powerful Role of Mucus

The first line of defense against pathogens in Anthozoans is mucus, a dynamic polysaccharide protein lipid complex, which covers the animal’s body.

Mucus is involved in several biological processes such as locomotion, structural support, food particle trapping and defense against multiple environmental stresses, predators, as well as infectious agents, parasites, and pathogens and environmental stress.

The ectodermal cells on the animal’s eyelashes contribute to the mucus transport of foreign particles towards the mouth of the polyp, where they are digested by swallowing.

Cytotoxic substances may also be present in the mucus, acting defensively and aggressively towards organisms of the same genus [105].

An active cytolysin and aliphatic antibiotic compounds have been isolated from the mucus of *Heteractis magnica*. The mucus from *A. equina* is composed of water, proteins, carbohydrates, lipids and inorganic matter [12,105,106] and in vitro, it deploys several activities including cytotoxicity vs. rabbit erythrocytes and tumor cell line k562. An antibacterial lysozyme-like activity has also been found. Lysozyme is an antimicrobial enzyme involved in internal innate defense systems. As a glycoside hydrolase, it catalyzes the hydrolysis of 1, 4-beta-linkages between N-acetylmuramic acid and N-acetyl-D-glucosamine in peptidoglycans component of Gram-positive bacteria compromising the integrity of bacterial pathogens through the lysis of the cell wall. The presence of an antibacterial activity together with a hemolytic and cytotoxic activity in *A. equina* mucus indicates its involvement in the defense system of this species against pathogenic invasion. Lysozyme-like proteins have already been found in other sea anemones [107].

### 7.2. Cellular Responses

#### 7.2.1. Phagocytosis

Circulating invertebrate immune cells are capable of phagocytosis, engulfing and destroying foreign invaders. Innate immune systems also include recognition proteins that bind to molecules of the bacterial wall, fungi and other pathogens, and recognition of foreign agents triggers the production of antimicrobial peptides stored in the cells [104]. Within the Cnidaria, cells with phagocytic activity toward potential pathogen substances were found in the sea anemone *A. equina* and in the soft coral *S. exserta* [108,109].

Cells specialized in the phagocytosis process against the *Aspergillus sydowii* fungal pathogen have been identified in the *Gorgonia ventalina* [110]. In the gorgonaceae cells, phagocytosis is activated post trauma and thermal stress [111]. Olano & Bigger [109] classified phagocytes in granular amoebocytes, epidermal cells, sclerocytes, mesogleal cells, and gastrodermal cells. 

#### 7.2.2. Cytotoxicity

**Figure 3:**
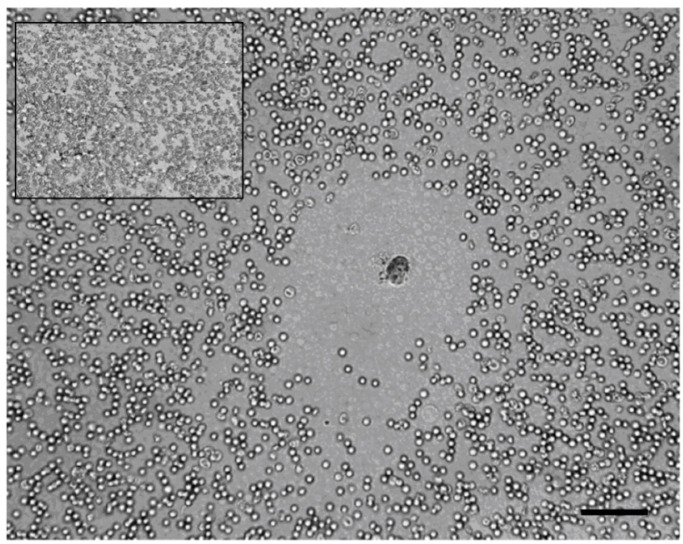
Plaque of lysis assay from cells isolated from *Actinia equina* against rabbit erythrocytes. Lysis plaques in a Cunningham–Szenberg chamber were observed when cells were mixed with target erythrocytes. Granulocytes were cytotoxic cells. Scale bar: 100 µm.

*A. equina* toxins have been found in specific marginal outgrowths of the body-wall (acrorhagi) and in cellular components of gastrodermal fluid [108] such as equinatoxins and sodium and potassium channel peptide toxins [112,113]. From internal liquid in *A. equina* specimens, six cell categories have been characterized and their lytic activity against several mammals’ erythrocytes types was shown [114]. In Figure 3, the plaque lysis assay of cells of separated bands from a Percoll gradient, applied on a cellular suspension of *A. equina*, is shown by phase-contrast microscope. 

Contrary to the belief that nematocysts are the only organ capable of releasing toxins with specific biological activity, some types of cells identified as granulocytes in *A. equina* contain lytic molecules. In some cnidarian species, amebocytes participate in the wound healing process [110] as with the hard-coral *P. cylindrical*. Coagulation leading to clot formation occurs via the degranulation of immune cells and the incorporation of cellular debris and extracellular matrix followed by infiltration of immune cells into tissues in the wound area.

In some invertebrates, a phase characterized by the infiltration of amoebocytes and the presence of fibroblasts able to control extracellular matrix production and collagen release was identified. The epithelial cells proliferate and migrate to regulate the re-epithelialization process [111].

#### 7.2.3. Reactive Oxygen Species (ROS) and Antioxidant

Reactive oxygen species (ROS) are free radicals derived from oxygen or nitrogen, involved in cellular reduction–oxidation (redox) reactions and counterbalanced by antioxidant compounds and enzymes especially in conditions of environmental stress.

At low doses, ROS act as signaling molecules, such as in immune response and apoptosis, while at higher doses they produce oxidative stress dangerous for cell components of both host and pathogens, such as nucleic acids, proteins, and lipids [115].

ROS are released by phagocytic cells or mobile amebocytes to help kill pathogens. During respiratory bursts in Anthozoans, hydrogen peroxide (H_2_O_2_) can be used to destroy pathogens or as by-products of melanin and oxidase enzymes formation [116].

Robb et al. [117] showed that the phagocytes in the mesoglea of the anemone *A. equina* release chromatin that may combine with the mucus to trap microbes subsequently destroyed by the activity of AMPS or ROS. Nitric oxide (NO) was produced in response to LPS in *Aiptasia pallida*, while nitric oxide synthase (NOS) has been revealed in *Aiptasia pallida, Acropora millepora*, and *Lobophytum pauciflorum* [118].

Elevated temperature and high irradiance can destroy the chloroplast and photosynthetic apparatus to start the bleaching cascade, considered a host innate immune response to a compromised algae, much like innate immune responses in other host–microbe interactions. ROS production by damage to both photosynthetic and mitochondrial membranes under stress conditions activates the caspase pathway. This promotes loss of symbionts and the innate immune response by NF-κB that activates apoptosis directly or induces the expression of inducible nitric oxide synthase (iNOS) that produces nitric oxide (NO). The combination of NO with O_2_—causes a mitochondrial membrane damage triggering caspases activation and apoptosis. [118].

To prevent oxidative stress, the cell uses various enzymatic and non-enzymatic antioxidants as nitrogen. Enzyme-based antioxidants are scavenging molecules stable enough to donate an electron to ROS, thus reducing its capacity to damage. These antioxidants delay or inhibit cellular damage mainly through their free radical scavenging property. Superoxide dismutase (SOD) catalyzes the dismutation reactions of the superoxide radical into hydrogen peroxide converted to a harmless molecule by catalase (CAT) and peroxidase in molecular oxygen and water. Organic hydroperoxide undergoes degradation through the peroxidase glutathione (GSH-Px). Nonetheless, over-production of ROS results in lipid peroxidation of polyunsaturated fatty acids (PUFAs), damages in the DNA molecules, and protein oxidation states [119].

Anthozoans possess many types of enzymatic antioxidants, peroxidases [120], SOD [121] and CAT [122]. This antioxidant activity varies with alteration in environmental conditions and particularly during bleaching or in response to both injury and infection.

*Anemonia viridis* has two kinds of SOD (CuZnSOD and MnSOD) enzymes, both of which are found in the ectoderm and the endoderm of this anemone. In this species, as in the coral *Goniopora stokesi*, the antioxidant molecules are associated with granular vesicles and accumulation bodies in the peroxisomes [123].

Wounds and alteration to the environmental temperature modulate a production of antioxidant enzymes in Gorgonians species such as *P. elisabethae*, *P. americana*, *Eunicea fusca* and *L. chilensis*. Thus, enzymes act as biomarkers for environmental stress assessment [124].

In *Stylophora pistillata* [125], the production of antioxidant enzymes is correlated with the bleaching process, demonstrating the strong involvement of light radiation and thermal stress in the ROS activation. 

The symbiotic zooxantellae of hermatypic or reef-building corals such as *Seriatopora hystrix* and *Stylophora pistillata* produce a large amount of ROS. This production is followed by the activation of the caspase cascade up to apoptosis and bleaching of the polyp host [126]. 

The family of peroxidase enzymes, a subgroup of oxidoreductase, is involved in a number of defense and metabolic processes. In addition to the relationship with the formation of ROS, they attend in the detoxification of oxygen forms, phagocytosis activity, and melanin synthesis [127,128].

In *G. ventalina*, five peroxidase isoforms production is induced by inflammatory conditions due to infection with fungal pathogens, as demonstrated by Mydlarz and Harvell [120]. Peroxidase was localized in granular amoebocytes during the process of phagocytosis in the gorgonian *Swiftia exserta* [109]. In anemones, there are other molecules that contribute to antioxidant systems, but these are not scavengers—rather, they are fluorescent proteins (FPs). These are involved in photoprotection and eliminate hydrogen peroxide [129].

## 8. Inflammatory Processes in Model *Anemonia viridis*

Actiniaria is one of the most successful and diverse taxa of Anthozoa. Sea anemones occupy all marine habitats, depths and latitudes, despite their body simplicity [130]. The group comprises 48 families, 269 valid genera and more than 1000 species [131,132].

**Figure 4:**
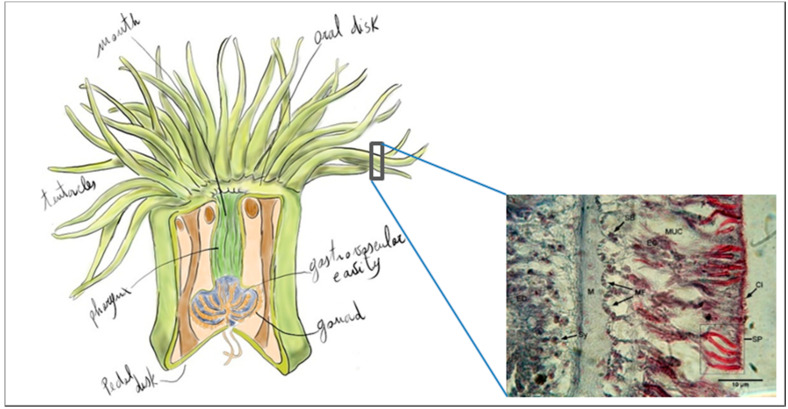
Anatomy of *Anemonia viridis*. Gomory stain of tentacles histological section (M: Mesoglea, Sy: Symbiont, Sp: Spyrocysts, Muc: Mucocytes, Ci: Cilia, MF: Muscular fiber). Bar: 10 µm.

*Anemonia viridis* is a symbiotic sea anemone from a wide geographical range of temperate areas, Its immense ecological success is due to a symbiotic association based on nutritional exchanges with zooxanthellae that live within endodermal cells facing the gastroderm and separated from the external environment by the ectodermal layer and the mesoglea. The tentacles are lined with venomous stinging cells called spirocysts used to paralyze the preys while the movement in the tentacles brings the food through the mouth for extracellular digestion (Figure 4).

From June through August, the oviparous snakelocks anemone reproduces sexually. In addition, longitudinal fission occurs: a literal sea anemone cleavage which starts from the basal disk. The whole process lasts from five minutes up to two hours [133]. 

*A. viridis* is a model for studies on symbiosis and environmental stress (temperature, light, symbiosis breakdown), power of acclimatization to climate change, evolution of innate immunity, and inflammatory responses [134,135].

**Figure 5:**
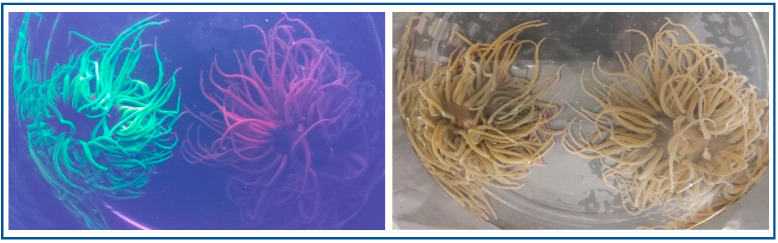
*A. viridis* color morphs based on pigment content. Specimens collected along the North Sicilian coast and maintained in the laboratory. The red (*rustica* variety) and green (*viridis* variety) pigment leakage is detectable after irradiation with ultraviolet light.

Different host genetic lineages, differing in their associated symbiont populations, have been identified. The species *A. viridis* is present in the Mediterranean with the following color morphs based on pigment content: *rustica, vulgaris, viridis, smaragdina* and *rufescens* [136]. These not only differ for morphology, reproduction modality and protein composition, but also with the presence of two green fluorescent pigments (gp499; gp522), an orange fluorescent pigment (OP) and a red-pigment (RP) non-fluorescent in the ectoderm with UV protection function (Figure 5).

These differences reflect an adaptation to environmental conditions, although they can also comprise explicit genetic differences divergent in the context of speciation phenomena.

In the northern coastal area of Sicily, we have previously characterized subtidal specimens that showed less pigment than infralittoral organisms, due to dysfunction or breakdown of symbiosis with zooxantellae in frequent swirling events, during which the temperature can increase considerably [137]. In anemone species, as with corals, bleaching is a stress response to environmental perturbation, including changes in salinity, increased visible and/or ultraviolet radiations, increased sediments, nutrients, or pollutants [138]. 

Studies have focused on identification of the etiological agents of coral diseases [139], but little work has focused on the cellular mechanisms that cnidarians possess to defend against pathogenic microbes. Anthozoans are potentially damaged by infections, anthropogenic stress, disease, and factors of climate change. In the last decade, the knowledge of recovery mechanisms after inflammatory events or injuries has increased. During prey catching, the Anthozoans species with soft tentacles, such as the Mediterranean Sea anemone *A. viridis*, can be subject to tentacle breakage, becoming vulnerable to pathogenic infections present in the environment. The immune humoral and cellular effectors trigger various inflammatory processes, which ensure the survival of the species in their natural environment [140].

Removal of microbial agents is carried out via phagocytosis and enzymatic activation to circumscribe the area of infection and inflammation [141]. This follows the activation of effective molecules by transduction of intracellular signals.

Sea anemones also use antimicrobial molecules from mucus as the host defense mechanisms against infectious disease. Their functional cellular immune system is analogous to vertebrate immune mechanisms [142].

Gene expression studies also suggest a conserved function for cnidarian innate immune pathways. In the coral *Acropora cervicornis*, several immunity related transcripts were shown to be upregulated in animals with white band disease including a C-type lectin, collectin, and a TLR-2 like gene [143]. In addition, challenge of the coral *A. millepora* with molecules of both viral and bacterial origins showed differences in expression of several immune genes and challenge with live pathogens lead to an increase in C3 expression [67]. Therefore, there is evidence for the involvement of cnidarian innate immune pathways, including the complement system, in the defense against pathogens.

Stress events or even natural contact with other individuals in the environment causes the rich mucus secretion of molecules with cytotoxic activity [144].

Furthermore, mucus from *Acropora palmata* inhibits water column marine bacteria [145]. Stronger antibacterial activity versus *Bacillus subtilis, Staphylococcus aureus, Salmonella typhimurium* and *Serratia marcescens* has been detected in specimens from populations living in stable environments and not stressed by the alteration of chemical and physical factors, especially temperature, than in samples isolated from bleached or stressed colonies [146].

Within scleractinian corals, several types of immune cells have also been identified, including granular amoebocytes, melanin-containing cells, chromophore cells, agranular (hyaline) cells and fibroblast-like cells in response to injury [147].

The protection role of amebocytes from injuries and infections in *A. equina* is demonstrated from ingestion of the gram-negative bacterium *Psychrobacter* [141]. In addition, granular amebocytes flow to the sites of fungal infections. In the soft coral *S. exserta*, phagocytic activity is dependent on the type of trauma, and normally the amebocytes are concentrated around inflicted wounds in order to clean up cell debris [105].

Palmer and Traylor-Knowles [148] described a study about the Caribbean gorgonian *Plexaurella fusifera* subjected to injury, during which amoebocytes and the photosynthetic endosymbiont form an epithelial front. The amebocytes, increased in number at the injured area, extrude mesogleal connective fibers to promote the regeneration process, while the zooxanthellae provide the energy used for the repair processes. 

In Anthozoa sea fan corals, a localized inflammatory reaction to fungal infection and a systemic response to environmental stress (such as seawater warming) triggered by an increase of granular acidophilic amebocytes normally involved in injury repair and a self/non-self-recognition system was described. Furthermore, along with the systemic cellular response, a related presence of melanin in sea fan tissue was detected. 

Particularly in Anthozoan tissues, reactive oxygen species increased following injury and infection events, and studying these can be useful for understanding the physiological dynamics during a shift from a functional to a not active host–symbiont interaction.

Disturbances such as anchoring, sedimentation, predation, and algal overgrowth result in wounding and compromised integrity of Anthozoan integrity. Disturbances that create wounds enable the entry of microbes into the tissues. It is thus important to determine how the species respond to wounding and physical damage and understand their capacity to regenerate the wounded areas.

Even for Anthozoans, the danger model theory can justify a series of humoral and cellular responses, such as inflammatory, so their immune system can distinguish between harmful and self-bodies. In soft corals and anemones, after an injury, the mesoglea swells and cells infiltrate from the mesoglea to the point of injury. Thereafter, a process of re-epithelialization begins from the margin of the wound.

In the sea anemone *Anthopleura elegantissima*, the repair area was detected at 72 h after thermal stressor treatment, and the movement of cells from the epithelium cells around and from the mesoglea was observed [149]. In the soft coral *P. fusifera*, re-epithelialization was detected 24 h after the onset of a wound [150]. Conversely, Vargas [151] did not find any specific reaction in *Montipora capitata* specimens, due to the narrow with of mesoglea.

Our research *A. viridis* innate responses after injection of various bacterial and non-pathogenic substances of varying size showed the reaction of a swelling of the pedal disc and the overlying portion of the body, as well as the formation of a small yellowish portion.

**Figure 6:**
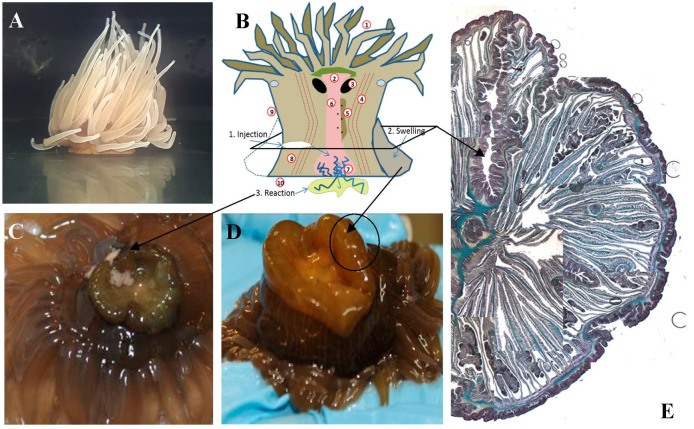
Morphology variation after bacterial infection in *A. viridis.* (**A**), Schematic model of anatomy and injection site, swelling and reaction (**B**), Reaction zone (**C**), rejection and swelling of animal body 24 h after injection of suspensions of various heat-killed bacteria in (inset) the reaction after *E. coli* injection (**D**), *A. viridis* Gomori stain histological section (**E**), The original figure was produced for the study published by Trapani et al., [135]. The modified figure is consistent with the topic of the review.

By histological analysis in the section of the infected animals, a specific reaction and extrusion in the pedal disk was observed, and mesenteric filaments oocytes were found to be coming from the pores of the *aconzie* extrusion. The reaction zone, formed after injection, decreased over time until the specimens had completely recovered (Figure 6).

During the early responses of the Anthozoans to pathogen agents or environmental stress, enzymatic activity (i.e., peroxidase or hydrolase) plays a focal role. In addition, differentiation of cells and nematocysts are controlled by a modification of the alkaline and acid phosphatase levels. Among these, only the peroxidase has been widely studied as a marker of regeneration in *Hydra* [152]. 

Mydlardz et al., [110] examined the role and inducibility of defense responses in the *Gorgonia ventalina* peroxidase enzyme superfamily to an *A. sydowii* fungal pathogen infection. Several isoforms of peroxidase in healthy colonies were found indicating the involvement of peroxidase in a plurality of physiological functions, such as the activation of signal cascade mechanisms of transduction, or activity against the fungal type employed.

In *A. viridis*, we analyzed the protease, phosphatase, and esterase activity following the injection of different bacterial strains. Results suggested a relationship between inflammatory process and modulation of enzymatic activities and in particular, a strong correlation between the animal’s inflammatory response and increased phosphatase and esterase in the tentacles and body extract infected with *E. coli* and *V. alginolitycus*.

## 9. Conclusions

Cnidarians possess components of the main pathways of invertebrate immunity. Receptors and pathways already identified indicate that these basal invertebrates are far from “simple” in their dealing with potential invading microbes and pathogens. However, there are numerous gaps in current knowledge, with a serious lack of functional component studies that can play a crucial role in immune responses.

The need to recognize and distinguish between symbiont and pathogenic microorganisms may be a selective force driving this diversity of PRRs in cnidarians. Immunological signaling cascades in the Cnidaria are among the most complex components and highly conserved within invertebrate innate immunity. Some of them are homologous with mammalian cascades. 

The onset and maintenance of symbiosis in host cnidarians are associated with the downregulation of immune-related pathways host immunity. In fact, bleached Anthozoan specimens are less susceptible to pathogen infection, supporting the idea that reduced immunity is part of obligate symbioses with zooxantellae. Symbiodiniaceae downregulates host immunity to allow its survival in the host, similar to what occurs in other symbiotic mutualisms.

The chemical arsenal of Anthozoans represents the optimal strategy for survival, mainly through the production of neurotoxins, but also with the use of antimicrobial defense molecules. Multifunctionality alone can represent an optimal survival strategy, allowing these animals to be active predators through the production of neurotoxins and to resist bacterial infections caused by the possible breakage of the tentacles through the functionality of antimicrobial peptides.

This work aims to expand knowledge related not only to the characterization of the effectors of innate immunity of Anthozoans, and to the various and multiple activities of antimicrobial toxins and peptides, but also to analyze the inflammatory response in Anthozoans as metazoan model systems. This may flesh out the scenario of the first stages of the evolution of immunity.

The study of the inflammatory response, which occurred following the inoculation of various substances in *A. viridis*, enabled the observation of specific reactions, especially following the inoculation of bacteria such as *E. coli*. This specific response, which ends in the appearance of a rejection zone, suggests an extrusion of the damaged material by the animal, probably via the pores of extrusion of the acoustical present in the pedal disc, a defense mechanism not yet found in other cnidarians.

A further study was also carried out in the form of a histological analysis for the characterization of the tissues and the rejection area.

Enzymatic activity, specifically protease, phosphatase, and esterase activity, was also analyzed, showing how the inoculation of the different bacterial strains, in particular *E. coli*, alters the expression of these enzymes. These data agree with the results of the studies on inflammation, suggesting a correlation between the appearance of the inflammatory reaction and the modification of the enzymatic activity.

Despite progress over the past decade in learning about cnidarian immunity, many studies are still needed to understand how key processes are translated from receptor to effector.

We are evaluating the possibility of studying empirically responses of hosts to specific stressors, such as pathogenic challenge.

Cnidarians are also important to study from an evolutionary perspective because they possess a surprising amount of complexity in their genomes and share many pathways with vertebrates that are not present in other invertebrate groups. Information on innate immunity pathways in cnidarians can provide insight into the evolutionary origins of mechanisms vertebrates use to respond to foreign microbes.

## Figures and Tables

**Table 1:** Principal pattern recognition receptors (PRR) and microbe-associated molecular pattern (MAMP) [67,68] involved in cnidarian immunity and symbiosis establishing.

PRR	MAMP
Toll-like receptor (TLRs) [2,17,61] Extracellular leucine-rich repeat (LRRs) [36,69] Intracellular domain, TIR domain activating NF-κB pathway [17,52,70,71] Integrin [72,73] Scavenger receptors (SR) [74]	Bacterial flagellin, Glycans, lipopolysaccharide (LPS), double-stranded RNA, Peptidoglycan (PG), Glycosylphosphatidylinositol (GPI) anchors
Lectins [52,75,76,77]	Glycolipids, glycoproteins
Nucleotide-binding oligomerization domain protein (NOD) [2,8,52,69]	Intracellular MAMPs, including LPS
